# Genome Sequence of a Human Norovirus GII.4 Hong Kong[P31] Variant in Hong Kong, China

**DOI:** 10.1128/MRA.01391-19

**Published:** 2020-01-16

**Authors:** Erin H. Y. Tse, Lin-Yao Zhang, Sin-Leung Lau, Martin Chi-Wai Chan

**Affiliations:** a Department of Microbiology, Chinese University of Hong Kong, Hong Kong, China; b Stanley Ho Centre for Emerging Infectious Diseases, Chinese University of Hong Kong, Hong Kong, China; c Li Ka Shing Institute of Health Sciences, Faculty of Medicine, Chinese University of Hong Kong, Hong Kong, China; KU Leuven

## Abstract

We report the nearly complete genome of a norovirus GII.4 Hong Kong[P31] variant (GII strain Hu/HK/2019/GII.4 Hong Kong[P31]/CUHK-NS-2200) that was detected in a patient with gastroenteritis in August 2019. The genome was sequenced by metagenomic next-generation sequencing and was found to have 92.8% nucleotide similarity to the closest GII.4 norovirus sequence in GenBank.

## ANNOUNCEMENT

Human noroviruses, belonging to the *Norovirus* genus and the *Caliciviridae* family, are a major cause of gastroenteritis worldwide (1), posing a significant risk to young children, the elderly, and immunocompromised individuals (2). Genetically diverse noroviruses are categorized into 10 genogroups, of which GI, GII, GIV, GVIII, and GIX are known to infect humans (3). Most notably, GII.4 strains have dominated norovirus outbreaks since 2002, with new epidemic variants usually emerging every 2 to 3 years, although the most recent new variant appeared in 2012 (4). Here, we report the nearly complete genome of a norovirus GII.4 variant designated GII.4 Hong Kong[P31].

This norovirus GII strain (Hu/HK/2019/GII.4 Hong Kong[P31]/CUHK-NS-2200) was detected by PCR, as part of our ongoing norovirus surveillance study, in a stool specimen collected in August 2019 from a 42-year-old female patient hospitalized with acute gastroenteritis in Hong Kong, China. A metagenomic next-generation sequencing approach was used to determine the complete viral genome, as we described previously (5). A 0.65-μm-filtered stool suspension was digested with DNase, RNase, and benzonase to enrich the virus by eliminating unprotected host and microbial nucleic acids. Viral RNA was extracted using the MagMAX viral RNA isolation kit (Thermo Fisher), and cDNA synthesis was performed with tagged random octamers and SuperScript III reverse transcriptase (Thermo Fisher), followed by second-strand cDNA synthesis using a Klenow fragment (TaKaRa). The cDNA was purified using 1.8× AMPure magnetic beads (Beckman Coulter, Inc.) and amplified using Phusion Hot Start II high-fidelity DNA polymerase (Thermo Fisher). A library prepared using the Nextera XT DNA library preparation kit (Illumina) was subjected to paired-end sequencing (2 × 75 bp) on the NextSeq 500 system (Illumina).

A total of 1,226,904 paired-end reads were generated. Reads were trimmed using Trimmomatic 0.36 (6), with a passing rate of 97.0%. Reference sequence mapping was performed using Geneious R9 against the reference strain GII.4 Sydney[P31] (GenBank accession number JX459908), of which 8.5% of reads were mapped to norovirus. Taxonomic classification using Taxonomer (7) revealed that norovirus was the only pathogenic mammalian virus detected, with the remaining 7.7% of viral reads being matched to plant viruses. Genome coverage was 95.1%, and the mean sequencing depth was 1,980×. The viral sequence was confirmed by *de novo* assembly using SPAdes 3.13.0 (8) in metagenomic mode and by full-length virus capsid Sanger sequencing (5). The two methods yielded consistent results. The terminal 5′ end of the genome, with no coverage, was determined by amplicon Sanger sequencing (with the first 22 nucleotides corresponding to the forward primer binding region), while the polyadenylated terminal 3′ end was sequenced by the rapid amplification of cDNA ends (RACE) PCR method, using a tagged oligo(dT) primer.

The nearly complete genome of our strain is 7,556 nucleotides long (GC content, 49%), excluding the poly(A) tail, and it carries a type P31 polymerase. The best BLAST hit in GenBank was a norovirus GII.4 Osaka[P31] variant from Japan (Hu/GII.4/Osaka3/2007/JP [GenBank accession number AB541323]), with 92.8% identity at the nucleotide level. Our strain shares 92.6% identity at the amino acid level with the closest GII.4 Osaka variant from Australia (Hu/GII.4/NSW390I/2008/AU [GenBank accession number GQ845369]) for the virus capsid, and this capsid was designated GII.4 Hong Kong.

The detection of a GII.4 Hong Kong[P31] strain in a severe case of gastroenteritis is atypical for a middle-aged individual without any known risk factors. Whether this variant emerges in the future to cause a major epidemic remains to be seen.

### Data availability.

The genome sequence of the norovirus GII.4 Hong Kong[P31] strain (Hu/HK/2019/GII.4 Hong Kong[P31]/CUHK-NS-2200) has been deposited in GenBank under accession number MN400355. The raw Illumina reads are available under BioProject accession number PRJNA588106.
